# What do general practitioners think about an online self-regulation programme for health promotion? Focus group interviews

**DOI:** 10.1186/s12875-014-0214-5

**Published:** 2015-01-22

**Authors:** Jolien Plaete, Geert Crombez, Ann DeSmet, Myriam Deveugele, Maïté Verloigne, Ilse De Bourdeaudhuij

**Affiliations:** Department of Movement and Sport Sciences, Ghent University, Ghent, Belgium; Department of Experimental-Clinical and Health Psychology, Ghent University, Ghent, Belgium; Department of General practice and Primary health care, Ghent University, Ghent, Belgium

**Keywords:** Health promotion, Physical activity, Healthy nutrition, General practitioners, Self-regulation, eHealth, Tablets, Computer tailoring, Feasibility

## Abstract

**Background:**

Chronic diseases may be prevented through programmes that promote physical activity and healthy nutrition. Computer-tailoring programmes are effective in changing behaviour in the short- and long-term. An important issue is the implementation of these programmes in general practice. However, there are several barriers that hinder the adoption of eHealth programmes in general practice. This study explored the feasibility of an eHealth programme that was designed, using self-regulation principles.

**Methods:**

Seven focus group interviews (a total of 62 GPs) were organized to explore GPs’ opinions about the feasibility of the eHealth programme for prevention in general practice. At the beginning of each focus group, GPs were informed about the principles of the self-regulation programme ‘My Plan’. Open-ended questions were used to assess the opinion of GPs about the content and the use of the programme. The focus groups discussions were audio-taped, transcribed and thematically analysed via NVivo software.

**Results:**

The majority of the GPs was positive about the use of self-regulation strategies and about the use of computer-tailored programmes in general practice. There were contradictory results about the delivery mode of the programme. GPs also indicated that the programme might be less suited for patients with a low educational level or for old patients.

**Conclusions:**

Overall, GPs are positive about the adoption of self-regulation techniques for health promotion in their practice. However, they raised doubts about the adoption in general practice. This barrier may be addressed (1) by offering various ways to deliver the programme, and (2) by allowing flexibility to match different work flow systems. GPs also believed that the acceptability and usability of the programme was low for patients who are old or with low education. The issues raised by GPs will need to be taken into account when developing and implementing an eHealth programme in general practice.

**Electronic supplementary material:**

The online version of this article (doi:10.1186/s12875-014-0214-5) contains supplementary material, which is available to authorized users.

## Background

Chronic diseases (heart diseases, stroke, cancer, chronic respiratory diseases and diabetes) are leading causes of mortality worldwide, representing 60% of all deaths. Healthy nutrition behaviour and sufficient physical activity play an important role in the prevention of these diseases. Moreover, it is estimated that 80% of the prevalence of heart disease, stroke and type 2 diabetes, and 40% of cancers may be prevented through intervention programmes that address risk factors such as lack of physical activity and unhealthy nutrition in adults [1,2].

A promising intervention method to motivate people to adopt behaviours such as physical activity and nutrition, is computer tailoring [3]. Applying algorithms to the answers that participants provide to a questionnaire, makes it possible to generate a computerized personal feedback [4,5]. That way, personalized advice about physical activity and healthy nutrition is quickly formulated, and may be used during health counselling [6,7]. Computer-tailored messages have shown to be more effective in improving health behaviour than generic information, and have led to positive outcomes in nutrition and physical activity [3,8]. However, despite the successes of these programmes, there is room for improvement.

First, most computer-tailored interventions target determinants deemed important during the early stages of behaviour change, such as attitude and knowledge [3,8]. Two recent systematic reviews about computer-tailored interventions argued to go beyond targeting the determinants of these early phases, and to more explicitly adopt self-regulation models of behaviour change [3,8]. According to Maes and Karoly (2005), self-regulation is a goal guidance process that enfolds over time, and may occur in different phases. The first phase, is a pre-action phase in which existing problems (e.g. low level of physical activity) are recognized and intentions and goals for behaviour change are formed. In the second, action phase, people try to pursuit their intended goals and in the last phase people evaluate their behaviour change and try to maintain their behaviour. When developing health behaviour change interventions, it is important to also target determinants in the action and maintenance phase (e.g. Action Planning, Maintenance self-efficacy, Recovery self-efficacy) by integrating self-regulation skills (e.g. goal setting, self-monitoring) [9,10].

Second, dynamic computer-tailored interventions have shown to be more effective in health behaviour change compared to static computer-tailored interventions [11]. In static computer-tailored interventions, only one assessment at the beginning of the intervention is used. In dynamic tailoring, different assessment moments are used, making feedback on behaviour change processes possible [11,12].

Third, most existing computer-tailored interventions are self-guided without direct contact with an expert or therapist [8]. Nevertheless, some studies have shown a larger effect when personal contact was added to the computer-tailored intervention [13-15]. A personal contact with GPs may well have advantages: GPs already play a role in the promotion of physical activity and healthy nutrition in adults [16,17], albeit that they report obstacles, such as lack of training and skills, lack of time, patient reluctance, other priorities in patient care, lack of resources, scepticism about efficacy and GPs perceiving other health professionals as better suited [16-23]. Also, patients consider their GP as a reliable source of information about nutrition and physical activity [16,17]. Thus, implementing eHealth programmes in general practice may have advantages. The computer-tailored intervention may take over some tasks of the GPs. Also, the tailored advice delivered by computer-tailored interventions can prompt and guide GPs to further counsel their patients [24]. Finally, the possibility of a direct and repeated contact with a GP may result in more tenacious goal engagement and maintenance of the patients. However, some obstacles may remain, or new obstacles may emerge. The results of two studies on the feasibility of a computer-tailored intervention to promote healthy behaviour through GPs are illustrative in this regard. In a first study of Sciamanna (2004), a laptop computer was installed in the waiting room of a general practice, and delivered a computer-tailored feedback about smoking and physical activity. The personal feedback was based on participants’ activity level, readiness to change, processes of change and self-efficacy [24]. Unfortunately, only one out of ten general practices was successful in implementing the programme in daily practice. GPs identified several barriers such as an inconsistency of the program with practice workflow, inexperience of the staff, technical problems with the programme and printer, additional time burden, and the length of the programme [24]. In a second study of Shakeshaft (2006), hand-held computers were used to implement a computer-tailored programme for the provision of screening and brief intervention for alcohol consumption, smoking status and general health (physical activity, emotional problems, usual activities and social activities). Data were collected on a hand-held computer in the waiting room of general practice prior to the consultation. After the screening, tailored feedback was immediately provided. When patients consented, the feedback was read and discussed with their GP during the consultation. About 55% of the participants actually started the programme on the hand-held computer, of whom 80% completed all questions, and of whom 89% agreed to discuss the feedback with their GP during consultation. The majority of patients rated the use of the hand-held computer as excellent (36%), very good (29%), or good (24%). In contrast to the previous study, the authors concluded that the use of computer technology in primary care was feasible [25]. However, in the study of Shakeshaft (2006) only one general practice was involved and not all patients were invited to participate. Also, GPs were not asked about the feasibility of incorporating the programme in daily practice [25]. Of note, these two studies did not target self-regulation skills such as planning, goal setting, problem solving and self-monitoring of behaviour.

To address the above mentioned shortcomings, we developed an eHealth programme, named “My Plan”. In “My Plan”, the theoretical background and delivery mode of existing computer-tailored programmes were adapted [26,27]. First, “My Plan” integrates the principles of self-regulation and goal setting. Second, a more dynamic delivery mode was created by using several assessment and feedback moments. Participants could go back to the website to re-assess their behaviour, to evaluate goal progress, and to receive further tailored feedback. Third, a direct contact with the GP was made possible, and the GPs were involved in the dissemination of the intervention. Consequently, we also involved GPs in the process of development, adoption and implementation of the intervention. The aim of this study was to explore the opinions of GPs about the feasibility of this eHealth self-regulation programme using focus groups. In the focus group interviews we asked questions about the theoretical background, the target group and the delivery mode of the eHealth programme ‘My Plan’. By involving GPs early on in the process, we aimed to reach higher levels of feasibility, and to solve anticipated barriers early on [24]. Such approach may lead to a programme that is not only tailored to the patients, but also tailored to the context and practice of GPs.

## Methods

### Pilot study

Before the actual study with focus group interviews, we conducted a pilot study in which 25 GPs were face-to-face interviewed about their opinions on the use of an eHealth programme and self-regulation techniques for health promotion in general practice. The results of this pilot study are reported in Additional file 1. In sum, GPs reported to be positive about the use of computer tailoring, action planning and goal setting for the promotion of health behaviour in their practice, and considered an eHealth programme useful. They also endorsed the idea that self-management and collaborative decision in health was worthy of further consideration. Nevertheless, doubts were raised on how to deliver the eHealth programme in general practice. Furthermore, the idea of using the programme only for primary prevention (using the programme for all healthy patients) was not endorsed. Based upon the results of this pilot study, an interview guide was developed to help direct the conversation toward the topics and issues in the focus groups (Additional file 2).

### Design and procedure

Seven focus group interviews were conducted to evoke discussion among GPs and to gather more in-depth information and suggestions. It is important to note that the participating GPs did not see the programme in operation. Prior to the focus group interviews, a presentation was given about health promotion, and, in particular, about how health promotion may be achieved via computer tailoring and self-regulation. Different methods (goal setting, action planning, implementation intentions, problem solving, and self-monitoring) to apply the principles of self-regulation in general practice were explained, and the role of GPs in prevention was emphasized. Finally, an initial outline of the eHealth programme was presented to the GPs using Table 1. After the presentation, the interview guide was used to ask open-ended questions. Questions were related to the content, the delivery mode, and the target group of the proposed programme (see Additional file 2). Focus groups consisted of between 6 to 8 GPs, and were led by a moderator and co-moderator. Conversations lasted on average 1.5 hour, and were audiotaped and transcribed. The study was approved by the Ghent University Ethics Committee.

**Table 1:** **Design concept of the eHealth programme**

**Step**	**Where**	**Description**
1: Computer tailored feedback	In the waiting room of general practice on a tablet	When patients come into practice they are informed by their GP or by the practice assistant about the eHealth programme. When patients want to use the programme, they receive the tablet on which they can fill in a questionnaire. Based on their answers, tailored feedback on patients’ health behaviour is automatically generated by the programme.
	OR	
	During consultation on a tablet	
2: Making an action plan	In general practice (when enough time)	Based on the feedback, patients can decide if they want to make an action plan. They can make this action plan in the programme on the tablet by reading tips and answering questions. Patients make an action plan by anticipating on difficult situations and by selecting goals and strategies.
	OR	Patients can also choose to make this action plan back at home by using the programme on a website on their own computer.
	At home (when not enough time)	
3: Feedback by GPs	In general practice	When patients received their personal feedback/made their action plan, they can discuss this with their GP during consultation.
	OR	After the action plan is made, patients can also choose to email the plan to their GP.
	At home	
4: Follow-up	In general practice	When patients come back to practice, they can discuss with their GP if they have reached their goals.
	OR	Patients are prompted by email and can evaluate their health goals online by filling in a questionnaire to evaluate if they have reached their health goals.
	At home	

### eHealth programme

The eHealth Programme was based upon available literature and own previous work (Spittaels (2007); Vandelanotte (2003)) in this area [4,25,28,29]. The eHealth programme was based on the principles of goal setting and self-regulation to increase the autonomy of patients to change their behaviour. To extend the choices for patients, several health behaviours (e.g. fruit, vegetables and physical activity) were included in the eHealth programme.

In what follows, the programme that was presented in the focus group interviews, is briefly described. The programme outline of ‘My Plan’ was illustrated during a presentation to the participating GPs using Table 1. In the online computer tailored programme patients can freely select a target behaviour amongst several options, and fill in an online questionnaire. The answers to this questionnaire are used to provide short feedback that informs participants whether they reached the health norms for the chosen behaviour, and whether there is room for improvement. After answering this questionnaire, participants receive a personal advice, they decide whether and how they want to change their behaviour, and develop their own action plan. Patients also have the option to discuss their feedback with their GP during the consultation. That way, patients’ personal advice can be used by GPs to talk about patients’ health behaviour. The idea was that GPs are key to motivate patients and to keep them motivated. GPs were also informed that patients can go back to the eHealth programme on a website, make or adapt their action plan, send their action plan to their GP and discuss their action plan in follow-up consultations. Two different ways of delivery were presented: (1) using a tablet in the waiting room or, (2) using a tablet during consultation. On the tablet, patients could fill in a questionnaire about a self-selected behaviour, receive tailored feedback and make an action plan to change that behaviour.

### Participants characteristics

GPs of the Local Quality Circle in East Flanders (Belgium) were contacted by phone and asked if they were interested in a presentation and discussion about a programme for health promotion in general practice. Contact information of these GPs was obtained via the National Institute for Health and Disability Insurance (NIHDI) of Belgium. Data were collected from a convenience sample of seven Local Quality Circle groups of GPs. In total, 62 GPs participated in the seven focus group interviews. The mean age of the participating GPs was 52 (±10.1) years and the mean number of years of experience was 25 (±10.5) years. Most (n = 38) participants were male (62%), 32 GPs (52%) worked in group practices and 30 alone (48%). A practice assistant was employed by 20 GPs (32%). Only 7 GPs (12%) had a computer in the waiting room and only 7 GPs (12%) had wireless internet in their practice.

### Data analysis

The data were thematically analysed via NVivo software in several phases [30]. First, a coding scheme was developed based on the results of the pilot study. This scheme consisted of three main themes (1) theoretical background, (2) delivery mode and (3) target group. The sub-themes were based on the model of Ampt et al. (2009) that lists the factors that affect preventive care of GPs [16]. Factors included were ‘the attitude of GPs’, ‘norms of GPs’ (e.g. social expectations) and ‘controlling factors’ (e.g. barriers or facilitating factors) related to the proposed theoretical background, the delivery mode and the target group of the eHealth programme. Second, two researchers (MV and JP) independently started coding, using a combination of axial coding and inductive coding. That way, other sub-themes that arose in the transcripts itself could also be added to form the final coding template [30,31]. Disagreements were discussed by the two researchers until consensus was reached and interrater reliability was good (single measures ICC = 0.78). The final coding scheme was then used to code all transcripts. Finally, all codes of the transcripts were compared and interpreted [30-32]. The main aim of the analysis was to identify factors about content, delivery mode and target group that would need to be taken into account when developing the eHealth programme.

## Results

Quotes of GPs participating in the different focus groups are integrated in the text below and in Additional file 3, an overview of more illustrative quotes is given per theme and subtheme. In Table 2, an overview of solutions for the reported barriers is given.

**Table 2:** **Summary of solutions to deliver the eHealth programme in general practice**

**Barriers**	**Solutions**
Lack of time	• Let patients use the eHealth programme on a tablet during the waiting time before consultation.
	• Let patients start the eHealth programme in practice. When time is up let them halt the programme and motivate them to resume it back at home.
	• Give an additional flyer to patients to motivate them to resume or start the eHealth programme at home.
Risk of theft of the tablet when used in the waiting room.	• Use a security system in the waiting room.
Playing games on the tablet in the waiting room instead of using the eHealth programme.	• Use an application blocker on the tablet.
Not clear where the tablet is meant for.	• Use attractive posters and flyers that explain what the tablet is aiming for.
	• *For group practices:* let the practice assistants explain the eHealth programme to patients and let them motivate and assist patients to use the tablet.
Working with an appointment system, implicating there is no waiting time before consultation.	• Give the tablet after the consultation and let patients use it in the waiting room.
	*In case patients cannot stay in practice*, give an additional flyer with the web link on to motivate them to start the intervention at home.
Difficult to mention the eHealth programme because patients have other priorities.	*Examples of types of consultations in which the eHealth programme can be mentioned easily:*
	• When prescribing new medication;
	• When taking blood tests;
	• When giving vaccinations;
	• When prescribing anti-conception;
	• When patients have questions or start talking about nutrition and physical activity.
Emails for follow-up are too time consuming and create issues of responsibility.	• Use online follow-up modules based on computer tailoring.
	• Use a medical platform to receive the action plans of patients’.
	• Plan additional consultations with patients’ who want to discuss their advice and action plan.

### The theoretical background of the eHealth programme

The majority of the GPs were positive about the use of the principles of goal setting, self-regulation, and empowerment. According to the GPs, the proposed methods and techniques to apply the self-regulation principles may effectively lead to behavioural change. GPs supported the idea that patients have to decide themselves which behaviour they want to change, and to select their own health goals about physical activity and nutrition intake:

*“Patients choosing their own health goals is a conditio sine qua non for me.”*

Most GPs agreed that participants will be more likely to change their health behaviour when they make their own decisions about the extent of behavioural change compared with priori behavioural targets (health norms). GPs thought that patients would be more engaged in behaviour change when they set their own health goals:

*“In my opinion the good thing of this programme is that people can choose what is relevant for them. Because, when we tell patients what to do, we automatically get resistance of the patients.”*

*“I think patients’ motivation will be higher when they choose their own goals compared to when GPs impose health norms.”*

Most GPs mentioned that they already used this approach and, when applied, experienced that it was successful:

*“But we already use this kind of principles, I never tell patients what to do, I always let them choose by themselves, I never tell them to lose weight, that works better.”*

Nevertheless, some GPs had doubts about the effectiveness of setting too ‘easy’ health goals or health goals that do not reach the health norms. They suggested that at least the health norms should be mentioned, and that some encouragement is needed to reach the health norms in term:

*“Extra feedback with the health norms that prevent chronic diseases, is also needed.”*

*“Health norms should also be mentioned on the website, so that people know the health norms.”*

When patients would define health goals that are too difficult or too easy to reach, GPs found it instrumental that the programme would provide appropriate feedback, and advice the participants to adapt their goal to a more attainable or challenging goal:

*“Would it be an idea to also include feedback on extremely difficult goals? For example, if someone who never runs sets a goal to suddenly go running every day. They should get the advice that this goal is probably too high for them, that they should take it slowly and first set some more attainable health goals.”*

*“There should be feedback provided for people who set extremely low or high health goals.”*

The majority of GPs appreciated that patients receive computer tailored feedback and that they can evaluate their own behaviour on the website. GPs were positive that written feedback and action plans would be brief and to the point:

*“The personal feedback is something I believe in.”*

*“The feedback they receive should be short, clear and to the point, if necessary with bullets, but no long text.”*

The use of implementation intentions and self-monitoring by the use of follow up modules were positively evaluated:

*“We also have to be realistic, we cannot provide follow-up for all our patients, no, there must be an integrated system in the programme that provides follow-up feedback on the website.”*

GPs also mentioned that there should be sufficient options for the patients in health behaviour choices and action planning. Most GPs believed that the programme can be effective in short but not long term:

*“I think this will only lead to short time effects, after six months the effects will disappear.”*

They also thought that the response rate in the follow-up modules would be low, although they stated that follow-up is very important and that repeated messages are necessary:

*“No, they will just look at the advice once and then it will be over, they will not go back to the website again.”*

*“I think it will be effective if they have the possibility to go back to the website and look at the advice again and again.”*

GPs found it important that they see patients making an effort to change health behaviour in order to continue preventive counselling:

*“It is important that we can recognize our patients’ efforts.”*

### The delivery mode of the eHealth programme

The majority of GPs did not want to use the tablet during consultation due to a lack of time. They also reported that patients had other priorities during consultation:

*“I do not think it is feasible to use this programme on a tablet during consultation, because this will take some time.”*

*“Using the tablet in consultation is not feasible, it is way too time consuming, because people will have an extra problem they want to talk about.”*

GPs stated that the tablet could better be used in the waiting room. However, barriers to use the tablet in the waiting room were the risk of theft if not secured and the chance that the tablet would be used for games rather than for the health programme:

*“Let us start with a first issue: if you put a tablet in the waiting room, it will be stolen.”*

*“Starting the tablet should be easy and also, there should be no other programmes available on the tablet, because otherwise children will use the tablet to play with it.”*

When using the tablet in the waiting room, it should also be clear what the tablet is meant for. Therefore, GPs suggested using a poster and flyers in the waiting room:

*“It should be very clear and visible that patients can use the tablet in the waiting room, this can be done by the use of a poster.”*

The idea that the programme could be halted and resumed at home was also approved by most GPs:

*“Or, we can start during the consultation and afterwards, follow-up can be provided on the website.”*

GPs who work with an appointment system did not think it was feasible to use the tablet in the waiting room. GPs also reported that it is easier to use this programme in the waiting room of a group practice accompanied by a practice assistant than in a solo practice. Another suggested solution was to let patients use the tablet after consultation:

*“When a patients comes in and registers at the reception, they should receive the tablet and an explanation about the programme by the practice assistant.”*

*“You can talk about it in the consultation and ask to use the tablet in the waiting room after the consultation.”*

Opinions differed about the option to email the action plan to the GP. Some GPs indicated that they did not want to receive action plans of patients by email because they would feel responsible to give feedback and monitor goal progress:

*“Receiving all the feedback of patients gives a problem of responsibility, if we have all the advices and do not read it and patients get problems… that is dangerous, we have to be careful.”*

Other GPs thought this was a good idea because they can evaluate and give feedback on their patient’s action plans:

*“The added value is the link with the GP. So, the action plans and personal advices should also be sent to us. Then we can do something with this information, we can talk about it with our patients.”*

Some GPs came up with the idea to use an existing online platform to send the action plan to the GP. Another suggestion was to integrate the tool in the medical software programmes of GPs:

*“The ideal scenario is that patients’ action plans and advices are automatically integrated in our medical programmes.”*

The idea that patients can send their action plan to friends and family was positively evaluated. The opinions differed about whether the action plan should be printed in general practice or at the patients’ home:

*“Our practice will look like a printing business.”*

There were also GPs who preferred to work with flyers with a web link to the programme, instead of a tablet. Also the combination of both flyers and tablets was suggested by many GPs. They stated that it depends on the situation whether flyers or a tablet can be used:

*“Would it not be easier to just use paper documents like flyers?”*

*“I do not think this is an if-if story in which you can only use one method, it should be an and-and story in which you can use a tablet or a flyer according to different situations.”*

GPs stated that their role in this programme should mainly be to motivate patients and to suggest using the programme. Their role should be to make the tablet available in practice by putting it in the waiting room, by giving the tablet or a flyer after a consultation, by putting the web link on their own website and by communicating about the programme with patients:

*“In my opinion this is a feasible method, having a tablet available, so we can suggest patients to work on their health and use the programme in the waiting room.”*

GPs reported that it would be important to communicate about the purpose of the tablet and/or flyers to their patients. GPs also reported that it is important to have the opportunity and time to introduce the intervention programme to their patients:

*“There will be a tablet in the waiting room, but still, we should tell and motivate patients where the tablet is meant for.”*

They reported that the tablet can facilitate preventive counselling but they still thought it was difficult to do this for all patients. According to GPs, possible moments to use the programme are: consultations for a new medication, for vaccines, for a doctor’s prescription for contraceptive pills and for blood tests:

*“When patients are sitting in our practice because they are sick, it is difficult to start talking about prevention.”*

*“It is easy to ask it for example to someone who wants a prescription for contraceptive pills.”*

Some GPs were convinced that they are the most appropriate counsellors to provide the programme, because of GPs’ high level of authority. They also stated that they have the expertise to give appropriate feedback. However, others mentioned that they did not have enough expertise for preventive counselling. Another possible health care worker that was proposed, is the pharmacist. Many GPs also considered media or schools as good channels to implement this programme:

*“As a GP you can use your authority to convince people to use the programme.”*

### The target group of the eHealth programme

Opinions differed about possible exclusion criteria. Some GPs believed that the programme could also be used for patients with diabetes, obesity, hypertension and chronic heart diseases. However, some GPs found it important that patients with chronic diseases would discuss the feedback and action plan with their GP:

*“Patients with diabetes our high blood pressure would also benefit from it but the advices should be adapted to it.”*

Another concern of GPs was that not everybody is able to work with electronic devices, especially older patients, patients with a lower educational level and patients not speaking Dutch. Solutions proposed by GPs are the use of clear slogans, pictograms and pictures. According to some GPs, the digital programme would also not be suitable for all patients, because some people cannot work with a tablet or do not have an email address:

*“I think it will be difficult and strange for people who never used a tablet before, especially for older people.”*

## Discussion

Self-regulation and empowering patients are recently considered as a preferred method for promoting health behaviour in primary care [33]. According to self-regulation models, health goals are more likely to be attained if goals are personally relevant, if individuals make specific plans on how to attain their goals, and if they receive support [9]. Previous research showed more goal ownership in participants who set their own health goals. Participants that pursue own health goals are also less likely to drop out of behaviour change programmes, compared to participants who get prescribed health goals [34]. However, many health promotion interventions only consist of communicating the health norms/guidelines to participants. The health outcomes are then selected and defined by researchers and health care workers instead of by the patients themselves. This study aimed to investigate the opinion of GPs towards an eHealth programme that integrates self-regulation principles.

The majority of GPs were positive about the methods of self-regulation that were used in the eHealth programme. In future health promotion interventions, GPs may well be willing to adapt the role of facilitators instead of problem solvers. That way, the GPs will create a freedom of choice for patients in selecting health goals, and patients can make autonomous decisions, and yet feel supported by a professional. In the current ‘My Plan’ project, the additional comments of GPs that facilitate the self-regulation approach will be taken into account in the further development of the intervention (e.g. giving feedback to select challenging but attainable goals).

Despite the fact that GPs were positive about self-regulation and confirmed the importance of health promotion, contradictory results towards the delivery mode were reported. Although GPs see themselves as a suitable health care provider to deliver the eHealth programme, they reported barriers such as a lack of time to actually deliver the eHealth programme, which is in line with previous studies [16,35]. However, a variety of solutions to overcome these barriers were reported (see Table 2). These solutions differed between GPs and work flows (e.g. working with or without an appointment system). These results indicated that GPs are best provided with several options. So, GPs may select the delivery mode that best fits in their working system, and best fits for different patients and circumstances. For example, some GPs found it more suitable to use the tablet before the consultation whereas others would rather give the tablet after consultation. There were also GPs who suggested using a combination of resources. Apart from the tablets, GPs also proposed to use flyers (containing the web link to the programme) for patients who are less confident with a tablet, or in case of insufficient time to use the tablet.

All in all, taking these options into account, may also create a freedom of choice in the GPs, who may then become more autonomously motivated to advocate the programme. Therefore, the eHealth programme ‘My Plan’ will make use of a combination of systems (tablets and flyers) and will give GPs the freedom of choice to apply the intervention in a way that best fits into their workflow.

Probably, not all options for delivering the programme will be equally effective. Some GPs believed that patients would not visit the website at home when a flyer simply mentioned it. This is attested by several studies [36,37]. In these studies, patients received an invitation letter from their GP to visit a computer-tailored website. Of the patients who received a letter in the study of De Cocker et al. (2012), only 6.2% actually visited the website [36]. Similarly, in the study of Woolf et al. (2006), only 4% visited the website [37]. In a Flemish study of Spittaels and De Bourdeaudhuij (2006), flyers were distributed to hospital visitors in two different ways to invite adults to visit a tailored physical activity website. One group was personally approached and received a flyer by a researcher [14]. The other group did not receive personal contact and could just take a flyer in the waiting room. More participants who were approached by face-to-face contact (46%) registered to the website in comparison to participants without personal contact (6%). This all suggests that simply providing flyers in the waiting room is not sufficient and that personal contact and motivation by GPs to visit the website are needed [14,36].

In ‘My Plan’ some solutions to anticipated barriers are implemented into the programme (see Table 2). An example regarding the barrier lack of time, is that the programme can be halted and resumed at any time. This means that patients can choose when to finalize the different parts of the intervention. For example, when there was not enough time in the waiting room, patients can stop the programme and resume it at home. When there is no time available to use the tablet, when there are no tablets available or when patients refuse to use a tablet, flyers with a referring website link can be used as an alternative. However, it should be emphasized that GPs then have to motivate patients to visit the website and that they should explain the aim of the website [14,36]. Most GPs did not want to send emails to patients for follow-up. Therefore, in ‘My Plan’, follow-up modules with computer-tailored feedback were integrated in the programme itself. However, it is important that participants can discuss their own goals with significant others [9]. During the focus group discussion, some GPs mentioned that they found it important to evaluate and give feedback on patients’ action plans. In the study of Hwang et al. (2012), primary care providers were also interested in receiving reports about patients from an online weight-loss programme, but they were also concerned about the time required to review and act on these reports [35]. Therefore, future research will need to identify practical solutions for follow-up by GPs. In the study of Unrod et al. (2007), the tailored feedback was printed and placed in the patient record by a practice assistant. That way, the GP could discuss the files with the patient during a next consultation [5]. This may be a solution for group practices that have a practice assistant but not for solo practices. Another solution of Shakeshaft et al. (2006) is that patients are automatically asked after receiving the tailored feedback on their behaviour whether they want to discuss this feedback with their GP. In case of permission, patients can be instructed to take the handheld computer with them in the consultation, to discuss the feedback with their GP [25]. During the focus group discussion of this study, some GPs came up with the idea to send the action plan to an online, medical platform or to integrate the programme in the medical programmes of GPs instead of emailing the action plan to the email address of the GP. Definitely, the use of integrating eHealth platforms for primary care is an important topic for further research.

Our eHealth programme was developed for healthy adults (18 to 65 years old) without chronic diseases (diabetes, cancer and heart diseases) (= primary prevention). However, GPs indicated that the programme should not be restricted for use in primary prevention. Similar results were found in the study of Nielen et al. (2010), in which a questionnaire about the working methods in primary prevention was administered [38]. The results of this study showed that preventive measures were mainly performed by the GP when a patient asks for it or when patients visit a GP for other complaints. Nielen et al. (2010) suggested that primary prevention in general practice should focus on patients at risk [38]. In our study, GPs indicated that the programme is suitable for healthy patients, patients at risk and patients with chronic diseases. Therefore, using the programme only for patients without chronic diseases was considered not optimal. Nevertheless, because health norms about nutrition and physical activity may differ and may be more complex for patients with chronic diseases, a stand-alone advice is not appropriate for such cases. The eHealth programme may be adapted so that it provides tailored feedback taking into account the health state of patients. It may however remain mandatory to discuss feedback and action plan with the GP first before the pursuit of the health goals.

GPs mentioned that the eHealth programme would not be suitable for all patients. They believe that some patients, especially elderly patients and patients with a low educational level, cannot work with a tablet or do not have an email address or a computer at home. Similar results were found in the focus group interviews with GPs in the study of Hwang et al. (2012). GPs in that study also thought that motivated, younger patients would respond more to an online weight-loss programme in general practice [35]. Previous research, in which the adoption of health-related ICT by older adults was investigated, suggested to keep health-related ICT simple and to demonstrate substantial benefits of the programme [39]. Some of the GPs suggested using pictures, symbols and brief slogans to make the programme more understandable. Therefore, the content of an eHealth programme must be developed as easy and as understandable as possible. These suggestions will be followed during the further elaboration of the eHealth programme. Also, the use of computers, internet and electronic devises (e.g. smartphones, tablets) have increased significantly during the past years, suggesting that in time more older people will be able to use eHealth programmes [40]. For patients with a lower educational level, the cost of mobile phones, tablets and internet connection especially leads to inequalities in accessing to these instruments [40]. So, when GPs offer these instruments in their practice, patients with a low educational level status can also get familiar with these devices. Previous studies have also shown that participants with low educational level positively evaluated computer tailored programmes, and these programmes were also effective in changing health behaviour of participants with lower education [27,41,42]. eHealth projects are in continuous growth and the use of medical applications by clinicians and health professionals is still increasing [40]. Because the importance of electronic devices in the medical field increases, it could be important that GPs start using eHealth applications. An acceptability and feasibility study of the ehealth programme in general practice will therefore be conducted. It will specifically be investigated if the ehealth programme is accepted and feasible for older participants and for adults with a lower education.

A strength of this study is that the opinion and the expertise of GPs are taken into account from the beginning of the development of the new ehealth programme ‘My Plan’. However, we did not involve patients in the development process of the eHealth programme. Therefore, it will be important to evaluate the newly developed programme with patients in the next phase. Another limitation of this study is that GPs only received a programme proposal and did not see the programme in operation to give more detailed feedback. It is suggested to present the programme again to GPs when the first version of the eHealth programme is available on tablet.

Further research to evaluate the feasibility, acceptability, reach, adaptation and the effect of the eHealth programme is needed, before implementing the eHealth programme in general practice.

## Conclusions

GPs considered the use of the principles of self-regulation (goal setting and action planning) for health promotion as a good method. Therefore, future health promotion interventions in which self-regulation techniques are applied can be used for general practice. Preferences for the delivery mode of an eHealth programme in general practice differ among GPs, arguing against the use of a standardized protocol for every practice and GP. Different ways to use the programme in general practice and solutions with different choices on how and when to use the programme must be offered to GPs.

To deliver health promotion in general practice, different barriers should be taken into account in designing and implementing eHealth programmes. Flexibility and choice for the delivery of the eHealth programme by GPs is necessary. The delivery mode (e.g. using a tablet) must be adaptable for different practices, patients and situations to overcome barriers. Solutions that were formulated in this study and can be used to implement an eHealth programme in general practice are listed in Table 2. In future research, it is also important to focus on the usability and acceptability for patients, especially for older patients and patients with a low educational level.

## Additional files

Additional file 1:
**Results of the pilot study: face-to-face interviews with 25 GPs.** Before the actual study with focus group interviews, we conducted a pilot study in which 25 GPs were face-to-face interviewed about their opinions on the use of an eHealth programme and self-regulation techniques for health promotion in general practice. The results of this pilot study are reported in Additional file 1. Additional file 2:
**Interview guide for focus group interviews with GPs, this file contains the questions of the interview guide used for the focus group interviews.**
Additional file 3:
**Illustrative quotes of GPs, Quotes of GPs participating in the different focus groups are integrated in this file.**
