# Supplementary material for: What do general practitioners think about an online self-regulation programme for health promotion? Focus group interviews
Source: BMC Fam Pract. 2015 Jan 22;16:3. doi: 10.1186/s12875-014-0214-5 (PMC4311516; doi:10.1186/s12875-014-0214-5)
Supplement: Additional file 1: — Results of the pilot study: face-to-face interviews with 25 GPs. Before the actual study with focus group interviews, we conducted a pilot study in which 25 GPs were face-to-face interviewed about their opinions on the use of an eHealth programme and self-regulation techniques for health promotion in general practice. The results of this pilot study are reported in Additional file 1. [file 12875_2014_214_MOESM1_ESM.pdf]

Additional file 1 – Results of the pilot study: face-to-face interviews with 25 GPs

**View of GPs regarding an eHealth programme for health promotion**	**n 'agree'**
"A new programme for the promotion of physical activity and healthy nutrition for GPs is useful"	19
"I would use an eHealth programme in my practice"	18
"An eHealth programme can easily be integrated in my practice"	12
"It is a good idea to give tailored advice to patients"	23
"I think it is good that patients make an action plan with their own goals"	19
"I would discuss patients' action plans via email"	6
"I would discuss patients' action plans in another consultation"	21
**Occasions when an ehealth programme for health promotion can be used**	**n 'agree'**
When the GP thinks it is necessary	16
When patients ask for it	19
When there is a related complain	14
For all the adult patients that come to practice	1
**Which delivery mode do you prefer?**	**n 'yes'**
Computer/tablet in the waiting room	3
Flyers with the weblink on	14
A combination of a tablet in the waiting room and flyers	8
**Hindering factors for the use an eHealth programme in general practice**	**n 'agree'**
"A lot of patients cannot work with a computer"	13
"Time in the waiting room is too short"	11
"Patients do not want an extra consultation for preventive counselling"	11
"There is not enough time to mention this intervention during the consultation"	10
"Patients cannot fill in a questionnaire by themselves"	9
"Working with appointments"	9
"I do not have enough time to apply this intervention during consultation"	9
"Patients will not go to the website at home"	5
"The programme is difficult to mention to patients"	3

Additional individual quotes of GPs
"I think it is a good idea that people can make an action plan with their own goals, otherwise they would not succeed anyway."
"I would rather give a flyer, because people like to receive something that they can take with them, especially older people."
"I think flyers are a good idea, to get people to start talking about their health status. But I would also use a tablet for people who don't have a computer at home."
"I do not think it is a good idea that patients send their action plan to me, I do not want patients to know my email address."
"I think I would use it only when there is a specific indication for it."
"A tablet is definitely needed, because solely giving a flyer with a website on it, will not work."
"I absolutely do not want to use a computer in the waiting room, the patients I see cannot work with it and time is too short."
