# Supplementary material for: What do general practitioners think about an online self-regulation programme for health promotion? Focus group interviews
Source: BMC Fam Pract. 2015 Jan 22;16:3. doi: 10.1186/s12875-014-0214-5 (PMC4311516; doi:10.1186/s12875-014-0214-5)
Supplement: Additional file 2: — Interview guide for focus group interviews with GPs, this file contains the questions of the interview guide used for the focus group interviews. [file 12875_2014_214_MOESM2_ESM.pdf]

Additional file 2: Interview guide for focus group interviews with GPs

Questions on the delivery mode

1. How would you deliver the eHealth programme in your practice?
    a. Which delivery mode would most GPs prefer?
2. What can be hindering factors when providing a tablet with the eHealth programme in the waiting room?
3. What can be positive factors related to this delivery mode (providing a tablet with the eHealth programme in the waiting room)?
4. What can be hindering factors when using a tablet with the eHealth programme during consultations?
5. What can be positive factors when using a tablet with the eHealth programme during consultations?
6. Will this eHealth intervention help you to deliver more preventive care?
7. Are there any other suggestions or opinions on the delivery mode of the eHealth intervention?

Questions on the target group

8. Would you use the eHealth intervention for all you patients?
    a. Can you give some examples for which patients you would use the eHealth intervention?
9. Will all your patients be able/be willing to use this eHealth intervention?
10. How do you think patients will react when you talk about the ehealth programme/give them the tablet?

Questions on the theoretical background (self-regulation)

11. What is your opinion about the freedom of choice that is given to patients (e.g. choosing health behaviour, stating own health goals, own strategies, …)?
12. Do you think patients will make their own action plan?
13. Do you think it is a good idea that patients can email their action plan to their GP?
14. Would you discuss patients' action plan during the consultation?
15. Do you think this intervention can be effective in changing patients' health behaviour?

Are there any other opinions/suggestions regarding this eHealth intervention?
