# Supplementary material for: What do general practitioners think about an online self-regulation programme for health promotion? Focus group interviews
Source: BMC Fam Pract. 2015 Jan 22;16:3. doi: 10.1186/s12875-014-0214-5 (PMC4311516; doi:10.1186/s12875-014-0214-5)
Supplement: Additional file 3: — Illustrative quotes of GPs, Quotes of GPs participating in the different focus groups are integrated in this file. [file 12875_2014_214_MOESM3_ESM.pdf]

Additional file 3 –Illustrative quotes of GPs

Theme	Sub-theme	Illustrative quotes
Theoretical background of the eHealth programme	Attitude	• "We can provoke patients to use the programme and make their own choices. In this way, the first step comes from the patient. If we take the first step and tell patients what to do, behaviour chance will be less effective compare to when patients make their own choices"<br>• "I think that patients' motivation will be higher when patients choose their own goals compared to when GPs impose health norms."<br>• "But we already use these principles, I never tell patients what to do, I let them always choose by themselves, I never tell them to lose weight"<br>• "In my opinion, the good thing of this programme is that people can choose what is relevant for them. Because, when we tell patients what to do, we automatically get resistance of the patient"<br>• "I think that patients' motivation is much better when motivation is intrinsic, when the motivation comes from themselves. It is much more difficult, when we impose things…"<br>• "Patients choosing their own health goals is a conditio sine qua non for me"<br>• "A patient can choose to eat one piece of fruit, but really, will this help to prevent diseases?"<br>• "I think it is true, people should take actions themselves, they should discover what they are doing wrong"

	• The personal feedback, that is something I believe in.
Controlling factors (facilitating or hindering factors)	• "Extra feedback with the health norms that prevent chronic diseases , is also needed." • "Health norms should also be mentioned on the website, so that people know the health norms." • "I think that the programme should include the possibility that people are reminded about their health goals and can adapt their goals, via email or via SMS for example" • "Would it be an idea to also include feedback on extreme difficult goals? For example, if someone who never runs states a goal to suddenly go running every day… They should get the advice that this goal is probably too high for them, take it slowly and first state some more attainable health goals." • "There should be feedback provided for people who state extreme low or high health goals." • "The feedback they receive should be short, clear and to the point, if necessary with bullets, but no long text they have to read…" • "The personal advice should be offered with slogans and icons" • "We also have to be realistic, we cannot provide follow-up for all our patients, no, there must be an integrated system in the programme that provides follow-up feedback on the website."

	Norms (GPs expectations)	• "Our role as a GP should be to introduce the programme to patients, to motivate them and to provide some follow-up but not too much, they should accomplish their goals by themselves… I think the role of GPs is to suggest, to offer…"<br>• "I think this will only lead to short time effects, after 6 months the effects will disappear"<br>• "No, they will just look at the advice and then it will be over, they will not go back to the website again"<br>• "I think it will be effective if they have the possibility to go back to the website and look at the advice again and again…"<br>• "It is important that we can recognize our patients efforts"
Delivery mode of the eHealth programme	Controlling factors (facilitating or hindering factors)	• "It should be very clear and visible that patients can use the tablet in the waiting room, this can be done by the use of a poster."<br>• "It would be easier when there is a practice assistant."<br>• "I do not think this is feasible in my practice because I do not have enough time to talk about this with patients."<br>• "The tablet will be stolen if it is not protected."<br>• "I would put the website link on my website"

- “Let us start with the first thing, you put a tablet in the waiting room, it will be stolen…”
- “There will be a tablet in the waiting room, but still we should tell and motivate patients where the tablet is meant for?
- “At the reception, when a patients comes in and register at the reception, then they should receive the tablet and an explanation about the programme by the practice assistant”
- “You can talk about it in the consultation and ask to use the tablet in the waiting room after the consultation”
- “The use of a tablet will lead to more success compare to something on paper, just by having a tablet in their hands, that makes it more fun.”
- “I think that a lot of people still prefer a paper flyer instead of a tablet”
- “Would it not be easier to just use paper documents like flyers?”
- “I do not think this is an if, if story in which you can only use one method, it should be an and-and story in which you can use a tablet or a flyer according to different situations.”
- “I do not think it is feasible to use this during consultation, because this will take some time”
- “Using the tablet in consultation is not feasible, it is way too time consuming, because people then have an extra problem they want to talk about”
- “In my opinion this is a feasible method, having a tablet available, so we can suggest patients to work on their

health and use the programme in the waiting room.”

- “Patients do not want this when coming to practice, they come for something else, because they have the flu.”
- “People who are waiting in the waiting room are sick, they do not want to take a tablet and fill in a questionnaire at that moment. Give them a flyer, then they can do it one week later, when they are cured, that will be much more effective.”
- “Using a tablet is something that could work in group practices, because they have a lot of patients and also they have more time compare to practitioners in a solo practice. It is not feasible in a solo practice.”
- “Starting the tablet should be easy and also there should be no other programmes available on the tablet, because otherwise children will use it to play with it.“
- “Or, we can start during the consultation and afterwards follow-up can be provided at the website”
- “The added value is the link with the GP. So, the action plans and personal advices should also be send to us. So, we can do something with this information, we can talk about it with our patients…”
- “Looking at all the action plans of patients gives a lot of extra work for us”
- “Receiving all the feedback of patients gives a problem of responsibility, if we have all the advices and do not read it and patients get problems… that is dangerous, we have to be careful…”
- “A solution is that the action plan can be send to our medical platform, in this way the information is shared

		but we are not required to answer”<br>• “Our practice will look like a printing business”<br>• “It would be easy to print the advices and give it to the patient”<br>• “It is easy to ask it for example to someone who wants a prescription for anti-conception pills.”<br>• “Those who come for vaccines will also use the programme, they already came for preventive actions..”<br>• “It would be easy to give it to patients that come frequently to the practice, for example for new medication.”<br>• “You can also refer to the programme when people come for a blood test, or for a further check on their situation, just like a blood test.”<br>• “I think you will reach more people through a pharmacist then in our waiting rooms.”<br>• “As a GP you can use your authority to convince people to use the programme.”
Target group	Attitude	• “Patients with diabetes our high blood pressure would also benefit from it but the advices should be adapted to it.”<br>• “Patients with diabetes should also have the opportunity to use the programme but they should discuss it with us.”

Controlling factors (hindering factors)	• But, you also have older people that can work with a computer!<br>• “In our practice we have a lot of immigrant patients that do not understand our language, they cannot use it?”<br>• “You have to be realistic and take into account the people who we work with.. a lot of patients with a low economic status come to our practice, they cannot work with a tablet or do not have a computer.”<br>• “I think it will be difficult and strange for people who never used a tablet before, especially older people.”<br>• “In my practice there are a lot of people who have a low educational level and who won’t be able to use this kind of programme.”<br>• “Older people cannot work with a tablet”
